# CD36 deficiency attenuates experimental mycobacterial infection

**DOI:** 10.1186/1471-2334-10-299

**Published:** 2010-10-15

**Authors:** Michael Hawkes, Xiaoming Li, Maryanne Crockett, Angelina Diassiti, Constance Finney, Gundula Min-Oo, W Conrad Liles, Jun Liu, Kevin C Kain

**Affiliations:** 1 Institute of Medical Sciences, University of Toronto, Toronto, Canada; 2 Department of Molecular Genetics, University of Toronto, Toronto, Canada; 3 Department of Medicine, University of Toronto, Toronto, Canada; 4 Sandra A. Rotman Laboratories, McLaughlin-Rotman Centre for Global Health, Toronto, Canada; 5 Department of Biochemistry and Centre for the Study of Host Resistance, McGill University, Montreal, Canada; 6 McLaughlin Centre for Molecular Medicine, Toronto, Canada; 7 Tropical Disease Unit, Toronto General Hospital, Toronto, Canada

## Abstract

**Background:**

Members of the CD36 scavenger receptor family have been implicated as sensors of microbial products that mediate phagocytosis and inflammation in response to a broad range of pathogens. We investigated the role of CD36 in host response to mycobacterial infection.

**Methods:**

Experimental *Mycobacterium bovis *Bacillus Calmette-Guérin (BCG) infection in *Cd36^+/+ ^*and *Cd36^-/- ^*mice, and *in vitro *co-cultivation of *M. tuberculosis*, BCG and *M. marinum *with *Cd36^+/+ ^*and *Cd36^-/-^*murine macrophages.

**Results:**

Using an *in vivo *model of BCG infection in *Cd36^+/+ ^*and *Cd36^-/- ^*mice, we found that mycobacterial burden in liver and spleen is reduced (83% lower peak splenic colony forming units, p < 0.001), as well as the density of granulomas, and circulating tumor necrosis factor (TNF) levels in *Cd36^-/- ^*animals. Intracellular growth of all three mycobacterial species was reduced in *Cd36^-/- ^*relative to wild type *Cd36^+/+ ^*macrophages *in vitro*. This difference was not attributable to alterations in mycobacterial uptake, macrophage viability, rate of macrophage apoptosis, production of reactive oxygen and/or nitrogen species, TNF or interleukin-10. Using an *in vitro *model designed to recapitulate cellular events implicated in mycobacterial infection and dissemination *in vivo *(i.e., phagocytosis of apoptotic macrophages containing mycobacteria), we demonstrated reduced recovery of viable mycobacteria within *Cd36^-/- ^*macrophages.

**Conclusions:**

Together, these data indicate that CD36 deficiency confers resistance to mycobacterial infection. This observation is best explained by reduced intracellular survival of mycobacteria in the *Cd36^-/- ^*macrophage and a role for CD36 in the cellular events involved in granuloma formation that promote early bacterial expansion and dissemination.

## Background

*Mycobacterium tuberculosis *(*M. tb*) infects an estimated 2 billion people worldwide and is responsible for the most deaths annually (1.6 million/year) of any single bacterial pathogen[1]. However, only 5 to 7% of infected immunocompetent individuals develop disease during their lifetime[2], demonstrating the critical role of host factors in the control of *M. tb*. The histological hallmark of tuberculosis is the granuloma, composed of an inner core of activated macrophages primed for intracellular killing by surrounding T-lymphocytes[2]. Cellular dynamics within the granuloma foster interactions between the innate and adaptive immune systems[3], but granuloma formation may also promote bacterial expansion and dissemination during initial stages of tuberculosis infection[4]. Recent work using quantitative intravital imaging of early granuloma formation in zebrafish embryos has demonstrated that macrophages internalizing mycobacteria undergo apoptosis and are phagocytosed by previously uninfected macrophages recruited to the granuloma, which then become infected. Granuloma formation may therefore promote mycobacterial infection by allowing for intracellular persistence and expansion of bacteria as well as systemic dissemination through egress of infected cells to generate new granulomas[4]. Thus, macrophages play a central role in host-pathogen interactions during tuberculosis, acting as both the primary phagocytic line of defense against *M. tb *as well as the intracellular niche for bacterial replication.

Alterations in macrophage function have been implicated as risk factors for mycobacterial infection, including defects in NADPH oxidase[5,6], the interleukin (IL)-12-interferon (IFN)-γ axis[7-10], natural resistance-associated macrophage protein-1 (NRAMP1) [11,12], and the vitamin D receptor[13]. However, variability in host susceptibility to tuberculosis is not fully explained by alterations in these molecular determinants, and other host factors are likely to play an important role[14,15].

Model systems using both *M. bovis *Bacillus Calmette-Guérin (BCG) and *M. marinum *have been extensively used to study the pathogenesis and immunology of tuberculosis, each of which has its advantages and limitations[16]. Murine infection with *M. bovis *BCG is a well-established experimental model system of disseminated tuberculosis[3,17,18]. Mice of the BCG-sensitive C57BL/6 genetic background serve as permissive hosts for mycobacteria and develop systemic infection following inoculation via the intraperitoneal route[3,17]. Mycobacterial replication occurs in multiple organs and is ultimately controlled by adaptive host immune responses[19], mimicking the course of primary human tuberculosis. *M. marinum*, a relatively rapidly growing mycobacterial species, is a close genetic relative of *M. tuberculosis*[20] that has been used to study the pathogenesis of tuberculosis[4,21-23]. *M. marinum *causes systemic granulomatous disease in ectotherms such as frogs and fish and peripheral granulomatous disease (fishtank granulomas) in humans[24]. *M. marinum *shares genetic determinants of pathogenicity with *M. tuberculosis*, such as the ESX-1/RD1 locus, which induces recruitment of new macrophages to nascent granulomas[4,25], and modulates phagolysosome maturation and the intracellular fate of mycobacteria[26,27]. As such, *M. marinum *has provided valuable insights into tuberculous disease using *in vivo *and *in vitro *model systems. We used BCG *in vivo *and BCG, *M. marinum*, and *M. tb in vitro *to model aspects of tuberculosis in order to dissect the role of CD36 in disease pathogenesis.

The cell surface glycoprotein CD36, present in many cell types including macrophages, has been implicated in a variety of cellular processes including fatty acid transport, regulation of angiogenesis, atherosclerosis, inflammation, and as a pattern recognition receptor mediating innate immune responses to a range of pathogens, including mycobacteria[28]. CD36 belongs to the class B scavenger receptor family, a group of phylogenetically conserved molecules involved in sensing a variety of microbial products and endogenous ligands. CD36 plays a physiological role in the recognition and clearance of apoptotic cells by professional phagocytes[29]. CD36 also acts as a co-receptor with the Toll-like receptor (TLR) 2/6 complex that binds diacylglycerides, such as lipoteichoic acids, and participates in innate sensing and the phagocytic clearance of *Staphylococcus aureus*[30,31]. Compared to wild type (*Cd36*^+/+^) mice, CD36-deficient (*Cd36^-/-^*) mice are more susceptible to experimental *S. aureus *infection, exhibiting higher mortality, increased levels of bacteremia, and multiple renal and cardiac abscesses[30]. CD36 participates in the uptake of and inflammatory response to other bacterial species including *Escherichia coli *and *Enterococcus faecalis *in model cell systems *in vitro*[32]. CD36 contributes to macrophage-mediated clearance of *Plasmodium falciparum *parasitized erythrocytes[33,34], and CD36 deficiency is associated with a dysregulated cytokine response and increased mortality in experimental animal models of severe malaria[33].

Recently, a genome-wide RNA interference screen of *Drosophila melanogaster *macrophage-like cells identified the CD36 homologue Peste (Pes) and the related mammalian class B scavenger receptors as important factors in the uptake of mycobacteria[35]. Furthermore, reversible alterations in the expression of CD36 on peripheral monocytes/macrophages have been observed in patients with active tuberculosis[36]. Based on the hypothesis that CD36 deficiency may alter host susceptibility to tuberculosis, we examined the role of CD36 in mycobacterial infection *in vitro *and in an experimental model *in vivo*. We show that *Cd36^-/- ^*mice have decreased mycobacterial burdens and reduced granulomatous responses after challenge with BCG. Furthermore, macrophages deficient in CD36 restrict the growth of multiple mycobacterial species *in vitro*. Taken together, our results suggest that CD36 deficiency confers relative protection against mycobacterial infection.

## Methods

### Mice strains and mycobacteria isolates

*Cd36*^-/-^, *Tlr2^-/-^*, *Tlr4^-/-^*, and *Irak4^-/- ^*and wild type control (*Cd36*^+/+^) C57BL/6 mice were bred and kept in the animal facility at the University of Toronto. Animal protocols were approved by the Animal Care Committee of the University of Toronto, and all experiments involving animals were performed in compliance with current University of Toronto guidelines. Mice 8-12 wk of age were used in all experiments. *M. tb *strain H37Rv (TMC no. 102), *M. bovis *BCG-Pasteur strain, and *Mycobacterium marinum *type strain 1218R (ATCC 927) were routinely grown at 30°C or 37°C in Middlebrook 7H9 broth (BD Biosciences; Franklin Lakes, NJ USA) supplemented with 0.2% glycerol and 10% OADC (Oleic Acid, Albumin, Dextrose, Catalase; BD Bioscience; Franklin Lakes, NJ USA) or on Middlebrook 7H11 agar (BD Biosciences) supplemented with 0.5% glycerol and 10% OADC. Infections in experimental animals were initiated by intraperitoneal injection of 1.5×10^7 ^BCG.

### Determination of mycobacterial density in infected mice

At different points during infection, mice were euthanized by CO_2 _inhalation and spleens, livers and lungs were collected from infected mice. Half of each organ was homogenized and plated on 7H11 agar (BD Biosciences; Franklin Lakes, NJ USA), and incubated for 21 days at 37°C for BCG colony counts.

### Histopathologic examination

After collection of spleens, livers and lungs from infected mice, half of each organ was preserved in 10% formalin, embedded in paraffin and processed in 5 μm sections. Sections were stained with H&E for histopathology and with Ziehl-Neelsen stain for acid-fast bacilli.

### Intracellular survival of mycobacteria in murine macrophages

Thioglycolate-elicited macrophages from *Cd36*^+/+ ^and *Cd36*^-/- ^mice were seeded in 12-well polystyrene plates (300,000 cells/well) and allowed to adhere for 24 hr. They were then co-incubated with mycobacteria at a MOI of 10:1 (*M. tb*), 10:1 (*M. bovis *BCG) or 1:1 (*M. marinum*) for 3 hr. Cells were washed and incubated in medium containing gentamicin (RPMI 1640, with 10% fetal bovine serum, and 2.5 mg/L gentamicin) at 37°C for *M. tb *and *M. bovis *BCG, or at 30°C for *M. marinum*. At different times (1, 3, 5 or 7 days) after infections, cell lysates of macrophages were prepared and plated on 7H11 medium (BD Biosciences; Franklin Lakes, NJ USA) and bacterial colonies were counted after incubation at 37°C for 21 days (*M. tb *and *M. bovis *BCG) or at 30°C for 7 days (*M. marinum*).

### Uptake of mycobacteria by macrophages

Differentially labeled intracellular and extracellular mycobacteria were imaged using fluorescent confocal microscopy following phagocytosis by wild type (*Cd36^+/+^*) and *Cd36*^-/- ^murine macrophages. Thioglycolate-elicited macrophages from *Cd36*^+/+ ^and *Cd36*^-/- ^mice were seeded on glass cover slips at a density of 125,000 cells per cover slip. *M. marinum *was incubated for 10 minutes with sulfosuccinimidyl-6-(biotinamido) hexanoate (NHS-LC-Biotin, Thermo Fisher Scientific; Rockford, IL) at pH 8.0 in order to biotinylate the bacterial surface. Biotinylated *M. marinum *was then co-incubated with murine macrophages for 3 hr at a multiplicity of infection (MOI) of 100:1. Extracellular *M. marinum *were labeled using streptavidin-conjugated tetramethylrhodamine (streptavidin-TMR, Invitrogen; Carlsbad, CA). Macrophages were fixed and permeabilized (4% paraformaldehyde for 20 min followed by 0.1% Triton X-100 in 5% milk for 20 min) and intracellular *M. marinum *were labeled with a second fluorophore, streptavidin-conjugated Alexa Fluor(c) 488 (Invitrogen). Images were obtained using spinning disk confocal microscopy (Zeiss Axiovert 200 equipped with a Hamamatsu Orca AG CCD camera and spinning disk confocal scan head, Volocity acquisition software). Control conditions included non-biotinylated *M. marinum *(negative control for fluorescent labeling) and 10 μM cytochalasin D (Calbiochem, Gibbstown, NJ) to inhibit phagocytosis[37].

Internalization of *M. marinum *by wild type (*Cd36^+/+^*) and *Cd36^-/- ^*murine macrophages was quantified with a flow cytometric technique. Biotinylated *M. marinum *(MOI = 100:1) was co-incubated with murine macrophages in suspension at a concentration of 10^6 ^cells/ml in RPMI 1640 with 10% fetal bovine serum for 3 hr. Extracellular mycobacteria were labeled with streptavidin-conjugated allophyco-cyanin (eBioscience; San Diego, CA), cells were fixed and permeabilized according to manufacturer's protocol using BD CytoFix/CytoPerm™ (BD Biosciences; Franklin Lakes, NJ USA), and intracellular *M. marinum *was labeled with streptavidin-conjugated Alexa Fluor(c) 488 (Invitrogen). Flow cytometric analysis was performed using FACSCalibur (BD Biosciences; Franklin Lakes, NJ USA) acquired with CellQuest (BD, San Jose, CA) software and analysed with FlowJo 8.7.3 (Tree Star Inc., Ashland, OR).

Quantitative measurement of viable internalized mycobacteria was performed *in vitro *following 3 hr co-incubation of bacteria and macrophages, allowing for phagocytosis without significant intracellular replication. Thioglycolate-elicited macrophages from *Cd36*^+/+ ^and *Cd36*^-/- ^mice were plated and co-incubated with mycobacteria at MOI of 100:1 for 3 hr. Extracellular mycobacteria and non-adherent macrophages were removed by repeated washing (3 times) with media containing gentamicin (2.5 mg/l) and remaining intracellular mycobacteria were harvested by scraping and lysis using 1% Triton X-100 (Sigma; St. Louis, MO). Cell lysate was plated for mycobacterial counts as described above.

### Electron microscopy

*Cd36*^+/+ ^and *Cd36*^-/- ^thioglycolate-elicited peritoneal macrophages were plated on glass coverslips, then incubated with *M. marinum *(MOI 100:1) for 6 hr at 37°C. Cells were washed and fixed in 2.5% glutaraldehyde, postfixed in osmium tetroxide, dehydrated with alcohol, and embedded in epoxy resin. Ultrathin sections were stained with uranyl acetate-lead citrate, then examined with a FEI Tecnai 20 transmission electron microscope with EDX, Gatan image filter, and 1 k by 1 k digital camera.

### Cell viability

*Cd36*^+/+ ^and *Cd36*^-/- ^peritoneal macrophages were plated in 96-well polystyrene plates (50,000 cells/well), and co-incubated with mycobacteria as above. After incubation for 1, 3, 5 or 7 days, 10% (v/v) MTS reagent (CellTitre 96 (r) AQ_ueous _One Solution Assay, Promega; Madison, WI) was added directly to culture wells, incubated for 2 hours, and the absorbance measured at 490 nm.

### Apoptosis assay

Fragmented DNA of apoptotic cells was end-labeled by a modified TUNEL assay (DeadEnd(tm) Colorimetric TUNEL system, Promega; Madison, WI) according to manufacturer's instructions and nuclei of apoptotic cells were identified on the basis of their darkly stained pyknotic nuclei. Caspase-3 activity was determined in cultures of macrophages co-incubated with mycobacteria according to manufacturer's instructions (Colorimetric CaspACE(tm) Assay, Promega; Madison, WI).

### Genetic polymorphisms in the vicinity of *Nramp1*

Genomic DNA was isolated from macrophages of *Cd36^+/+ ^*and *Cd36^-/- ^*using column purification according to manufacturer' instructions (QIAamp DNA blood mini kit, Qiagen, Valencia, CA). Five markers in the vicinity of *Nramp1 *(D1Mcg2, D1Mcg3, D1Mcg5, D1Mit19, D1Mit23)[38] were tested in *Cd36^+/+^*, *Cd36^-/- ^*mice (C57BL/6 genetic background) and in two reference strains (C57BL/6, BCG sensitive; and 129/Sv, BCG resistant). Four markers were polymorphic between the reference strains and all 4 showed that *Cd36^+/+ ^*and *Cd36^-/- ^*mice carried the C57BL/6 alleles.

### Production of reactive nitrogen and reactive oxygen intermediates

The Griess reaction (Griess Reagent System, Promega; Madison, WI) was used according to manufacturer's instructions to quantify the nitrite concentration in the supernatant of thioglycolate-elicited peritoneal macrophages (plated at a density of 200,000 cells per well in 96-well plates) co-incubated with mycobacterial (MOI = 10:1). Nitrite is a stable, non-volatile breakdown product of nitric oxide (NO), produced by activated macrophages as a mechanism for intracellular killing of mycobacteria.

Oxidative burst was assessed using a chemiluminescence assay. Adherent macrophages (200,000 per well in opaque 96-well plates) were stimulated with BCG (MOI = 100:1), 10 μM phorbol myristate acetate (PMA, Sigma; St. Louis, MO), or media alone in the presence of 100 μM luminol (Sigma; St. Louis, MO). Chemiluminescence was detected using a MonoLight 2010C luminometer (Analytical Luminescence Laboratory, San Diego, CA).

### Measurement of cytokine production

Blood was collected from euthanized mice by cardiac puncture, allowed to clot, and cleared by centrifugation. Serum was stored at -80°C and later assayed for cytokines using a cytometric bead array assay (Mouse Inflammation Kit, BD Biosciences) according to manufacturer's instructions.

For assays of cytokine production *in vitro*, macrophages were plated in 96 well plates at a density of 200,000 cells/well. Cells were washed, pre-incubated with IFN-γ (10 ng/mL) for 24 hours, then co-incubated with *M. marinum *or BCG over a time course of infection, at various multiplicities of infection. TNF and IL-10 concentrations in the culture supernatant were determined by commercial ELISA according to manufacturer's instructions (eBiosciences; San Diego, CA).

### *In vitro *model of cellular events in granuloma formation

Thioglycolate-elicited peritoneal macrophages from *Cd36^+/+ ^*and *Cd36^-/- ^*mice were seeded in 6-well polystyrene plates (1×10^6 ^cells/well) and allowed to adhere for 24 hr. Macrophages were then co-incubated with *M. marinum *(MOI = 10:1) for 3 hr at 37°C, and washed three times to eliminate extracellular bacteria. Cells were incubated overnight in RPMI 1640 and gentamicin (2.5 mg/l) to induce apoptosis by serum starvation. Control macrophages were incubated in RPMI 1640 and gentamicin (2.5 mg/l) with 10% fetal bovine serum. Apoptosis was confirmed by elevated capase-3/7 activity[39], determined using a commercially available kit according to manufacturer's instructions (Apo-ONE Homogeneous Caspase-3/7 Assay, Promega). Apoptotic cells containing mycobacteria were scraped and co-incubated with fresh macrophages, plated at a density of 1×10^6 ^cells/well in 6-well plates for 3 hr at 37°C. Macrophages were washed three times with media containing gentamicin (2.5 mg/l) to eliminate extracellular apoptotic cells and bacteria. Macrophages were then harvested by scraping and lysis using 1% TrotonX-100 (Sigma). Cell lysate was plated for mycobacterial counts as described above.

## Results

### *Cd36^-/- ^*mice restrict mycobacterial growth relative to wild type (*Cd36^+/+^*) mice

**Figure 1:**
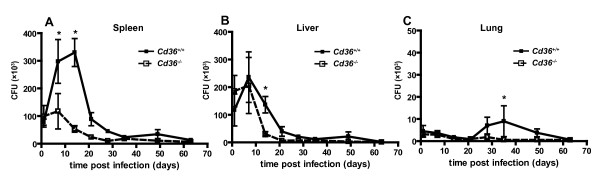
***Cd36^-/-^*mice have lower bacillary burden relative to *Cd36^+/+ ^*controls after challenge with *M. bovis *BCG**. **A**. Intraperitoneal infection (day 0) with BCG (1.5×10^7 ^organisms) resulted in a rise in mycobacterial counts in the spleens of *Cd36^+/+^*(wild type) mice to a maximum at day 14 with subsequent decline. The mycobacterial counts in *Cd36^-/- ^*mice (white box, dashed line) were lower overall (*p *< 0.001), and at specific time points (day 7, *p < 0.01; day 14, *p < 0.001). **B **and **C**. Differences between genotypes were less pronounced in the liver (**B**) and lung (**C**). Results are displayed as mean ± SEM, with 8 mice per group at each time point, representing two pooled independent experiments.

Based on observations implicating class B scavenger receptors in the uptake of *Mycobacterium fortuitum*[35], we hypothesized that disruption of the *Cd36 *gene would result in an altered host response to mycobacterial infection. To test this, we experimentally infected *Cd36^-/- ^*and *Cd36^+/+ ^*mice with *M. bovis *BCG and examined the burden of mycobacteria and histopathology over the course of infection. All mice survived BCG infection. Consistent with previous reports[17,40], we observed an initial rise in the BCG counts in the spleen, reaching a local maximum at 2-3 weeks after infection, with subsequent continuous decline over 9 weeks of infection (Figure 1). Of note, *Cd36^-/- ^*mice had lower BCG CFU counts in the spleen overall (*p *< 0.001) and at the peak of infection (day 14 post infection; mean ± SEM 5.4 ± 1.1×10^4 ^*vs *3.3 ± 0.5×10^5 ^CFU; 83% lower; *p *< 0.001, Figure 1A and 1D). Differences in total spleen bacillary load were attributable to both relative splenomegaly (i.e., greater average spleen weight, *p *< 0.001), as well as a higher density of BCG per gram of splenic tissue (*p *= 0.02) in *Cd36^+/+ ^*mice compared to *Cd36^-/- ^*mice. Significantly lower bacillary loads at peak (day 14) were observed in the livers of infected mice (*p *= 0.005), with a similar trend in the lungs (*p *= 0.054). Consistently, mice with higher mycobacterial counts in the spleen had correspondingly higher counts in the liver (Spearman's ρ = 0.818, *p *< 0.001).

**Figure 2:**
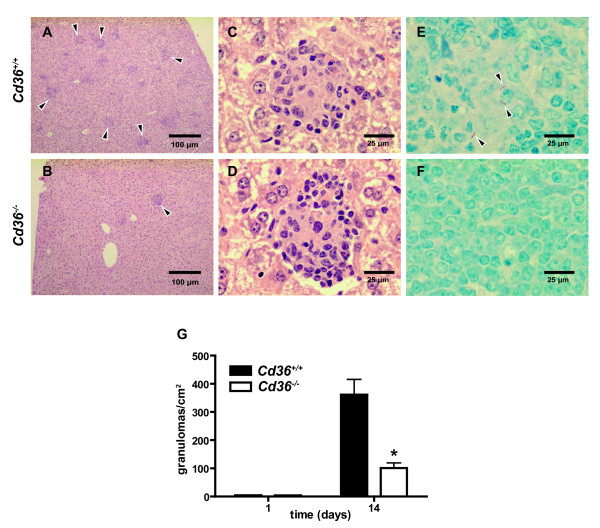
**Histopathological sections of organs of *Cd36^+/+ ^*(upper row) and *Cd36^-/- ^*(lower row) mice 14 days after IP infection with *M. bovis *BCG**. **A **and **B**. Liver sections (H&E stain, 10× magnification) demonstrate more numerous granulomas in *Cd36^+/+ ^*mice (arrowheads, **A**) compared to *Cd36^-/- ^*mice (arrowhead, **B**). **C **and **D**. Liver sections (H&E stain, 100× magnification) demonstrate similar microarchitecture of individual granulomas. **E **and **F**. Splenic sections (Ziehl-Neelsen stain, 100× magnification) show multiple acid fast bacilli (AFB) in a single field for *Cd36^+/+ ^*mice (**E**), and no visible AFB in *Cd36^-/- ^*mice (**F**). **G**. Liver granuloma cross-sectional density at day 14 post-infection was lower in *Cd36^-/- ^*mice (*p = 0.0038). All granulomas in each histopathological section were counted, with observer blinding, and normalized to the liver cross-sectional area. Data represent mean ± SEM, 4 mice per group.

On histopathological examination, there were fewer granulomas in the livers of *Cd36^-/- ^*mice compared to the *Cd36^+/+ ^*mice (Figure 2A and 1B), but the microarchitecture of the granulomas appeared unchanged (Figure 2C and 2D). In addition, fewer acid-fast bacilli were visible in splenic tissue sections of *Cd36^-/- ^*mice (Figure 2E and 2F), consistent with the lower BCG bacillary loads observed in *Cd36^-/- ^*mice. Quantitative assessment of liver sections demonstrated a 68% reduction in cross-sectional granuloma density in *Cd36^-/- ^*mice (median 116 (range 46 to 124) *vs *359 (239 to 489) granulomas/cm^2^; *p *= 0.029; Figure 2G).

### *Cd36^-/- ^*macrophages restrict mycobacterial growth *in vitro *relative to *Cd36^+/+ ^*macrophages

**Figure 3:**
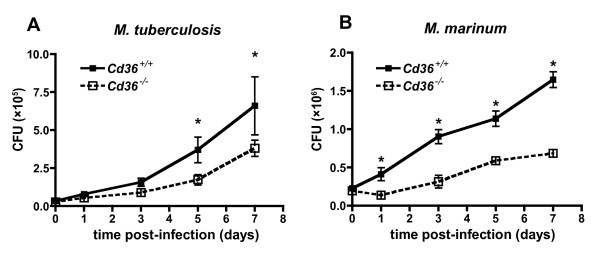
**Co-cultivation of mycobacteria with *Cd36^-/- ^*murine macrophages**. *In vitro*, mycobacterial infection of thioglycolate-elicited peritoneal macrophages resulted in lower mycobacterial loads in cultures of *Cd36^-/- ^*macrophages (white box, dashed line) compared with wild type controls (black box, solid line). **A**. Infection with *M. tuberculosis *(MOI = 10:1) produced a progressive rise in mycobacterial counts over 7 days, with significant difference (*p *< 0.0001) between groups. **B**. Similar results were seen after infection with the rapidly growing *M. marinum *(MOI = 1:1, *p *< 0.001). Data are shown as mean +/- 95% CI with 4 to 6 replicates at each time.

The macrophage plays a central role in host defense against *M. tb*, and several molecular determinants of host susceptibility to mycobacterial infection involve alterations in macrophage function[41]. In order to examine the mechanisms by which *Cd36^-/- ^*mice restrict mycobacterial infection, we exposed peritoneal macrophages derived from *Cd36^-/- ^*and *Cd36^+/+ ^*mice to *M. tb in vitro*. After infection with *M. tb*, intracellular mycobacterial counts progressively increased over 7 days, with fewer *M. tb *inside *Cd36^-/- ^*macrophages than *Cd36^+/+^*macrophages (*p *< 0.0001, Figure 3A). Mycobacterial counts were also lower overall in *Cd36^-/- ^*compared to *Cd36^+/+ ^*macrophages infected with *M. marinum *(*p *< 0.0001, Figure 3B) and BCG (*p *< 0.0001, data not shown). Because the intracellular growth restriction pattern of *M. marinum *was similar to *M. tb*, we used *M. marinum *as a model organism in subsequent *in vitro *experiments, replicating our findings with BCG where feasible.

### Mycobacterial internalization is similar in *Cd36^-/- ^*and *Cd36^+/+ ^*macrophages

Previously, the CD36 homologue Peste (Pes) in *D. melanogaster *as well as mammalian class B scavenger receptors have been implicated in the uptake of *M. fortuitum*[35]. Therefore, we tested the hypothesis that differences in the initial uptake of mycobacteria by macrophages might account for the differences observed in the mycobacterial bacterial loads following *in vitro *infection. However, using several experimental approaches including confocal fluorescent microscopy, flow cytometry, *in vitro *co-cultivation, and electron microscopy, we found no significant difference in uptake of mycobacteria by *Cd36^-/- ^*and *Cd36^+/+ ^*murine macrophages.

**Figure 4:**
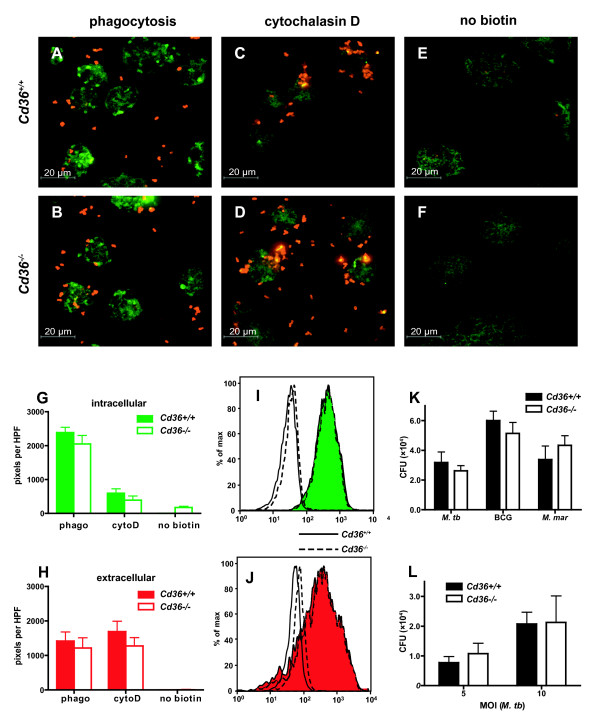
**No difference in uptake of mycobacteria between *Cd36^+/+ ^*and *Cd36^-/- ^*macrophages**. **A **to **F**. *M. marinum *was biotinylated and incubated with *Cd36^+/+ ^*(top row) and *Cd36^-/- ^*(bottom row) macrophages for 3 hours to allow phagocytosis. Extracellular *M. marinum *was labeled using streptavidin-conjugated tetramethylrhodamine (TMR) and appears red. Macrophages were fixed and permeabilized and a second streptavidin-conjugated fluorophore (AlexFluor488(c)) was used to label intracellular *M. marinum *(appears green). **A **and **B**. Intracellular (green) *M. marinum *is readily visible within *Cd36^-/- ^*macrophages, with uptake qualitatively equivalent to *Cd36^+/+ ^*macrophages. **C **and **D**. Control conditions using cytochalasin D (10 μM) to inhibit phagocytosis, demonstrating decreased intracellular (green) mycobacteria in both *Cd36^+/+ ^*and *Cd36^-/- ^*macrophages. **E **and **F**. Control conditions using unbiotinylated mycobacteria, demonstrating the specificity of fluorescent labeling for mycobacteria. **G **and **H**. Image analysis demonstrates similar quantities of intracellular (**G**, green) and extra cellular (**H**, red) *M. marinum *(p = 0.29) in *Cd36^+/+ ^*and *Cd36^-/- ^*macrophages. **I **and **J**. Flow cytometry showed no significant difference in the fluorescence intensity associated with intracellular (green) or extracellular (red) *M. marinum *between *Cd36^+/+ ^*(solid line) and *Cd36^-/- ^*(dashed line). Shown for comparison are negative control conditions (unbiotinylated *M. marinum*, no fill color). **K **and **L**. *In vitro*, *Cd36^+/+ ^*and *Cd36^-/- ^*macrophages showed similar uptake of three mycobacterial species, *M. tuberculosis *(MOI = 10:1), *M. bovis *BCG (MOI = 10:1) and *M. marinum *(MOI = 1:1) (*p *> 0.05 for all species). Similar uptake was observed at different multiplicities of infection with *M. tuberculosis *(*p *> 0.05 for each MOI).

Using differential labeling of biotinylated intracellular and extracellular *M. marinum *with distinct streptavidin-conjugated fluorophores before and after cell permeabilization (see Methods section), we qualitatively observed that *Cd36^+/+ ^*and *Cd36^-/- ^*macrophages internalize *M. marinum *to a similar extent (Figure 4A-F). Image analysis (Figure 4G and 4H) and flow cytometric analysis (Figure 4I and 4J) both showed that the uptake of *M. marinum *was quantitatively similar between *Cd36^+/+ ^*and *Cd36^-/- ^*murine macrophages.

Next, viable intracellular mycobacteria were enumerated after 3 hours incubation with macrophages and after repeated washing with media containing gentamicin to eliminate extracellular mycobacteria. Using three mycobacterial species, *M. tb, M. bovis *BCG and *M. marinum*, we found no significant difference in the mycobacterial counts between *Cd36^-/- ^*and *Cd36^+/+ ^*macrophages (Figure 4K), indicating that uptake of mycobacteria was similar in the presence or absence of CD36. We exposed macrophages to different inocula of *M. tb *and again observed no difference between *Cd36^-/- ^*and *Cd36^+/+ ^*macrophages in the uptake of mycobacteria at various multiplicities of infection (MOI) (Figure 4L). Experiments with *M. tb *at MOI = 10:1 were repeated four times to confirm this observation.

**Figure 5:**
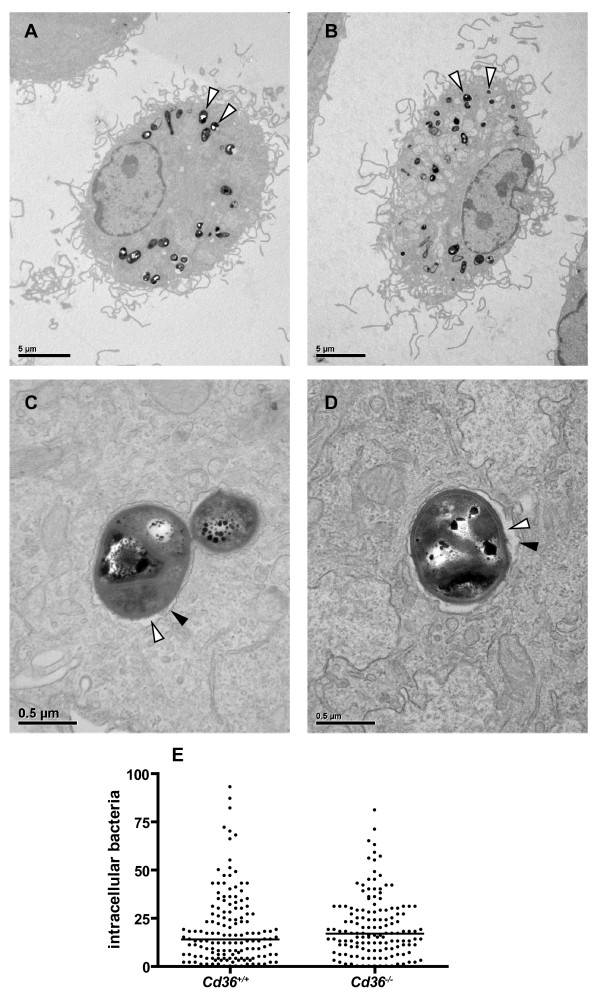
**Electron micrographs demonstrating similar numbers of internalized *M. marinum *bacilli after 6 hours co-incubation with *Cd36^+/+ ^*and *Cd36^-/- ^*macrophages**. **A **and **B**. Full cell view demonstrating electron-dense bacilli (arrowheads) within macrophages. **C **and **D**. In both cell types, bacilli are contained within a membrane- (black arrowhead) bound phagolysosome (white arrowhead, electron-luscent phagolysosome lumen). **E**. Enumeration of internalized bacilli demonstrated no significant difference in uptake of *M. marinum *between *Cd36^+/+ ^*and *Cd36^-/- ^*macrophages (*p *= 0.19). Bacilli were counted in a total of 293 macrophages from 3 independent experiments, taking a minimum of 30 random EM fields for each genotype in each experiment.

Finally, *Cd36^+/+ ^*and *Cd36^-/- ^*macrophages were co-incubated with *M. marinum *(MOI = 100:1) for 6 hr and visualized by electron microscopy. Numerous electron-dense bacilli were evident within cells of either genotype, and were contained within a membrane-bound phagolysosome (Figure 5A-D). Quantification of mycobacteria within a total of 293 random macrophages (3 pooled experiments) revealed no difference in the number of internalized *M. marinum *(Figure 5E; median (range) 14 (0-93) *vs *17 (0-81); p = 0.19) between *Cd36^+/+ ^*and *Cd36^-/- ^*macrophages.

### Cell viability and rate of apoptosis of *Cd36^-/- ^*and *Cd36^+/+ ^*macrophages are similar

CD36 has been implicated in macrophage apoptosis[36,42], which may be an important host defense strategy for the containment of intracellular mycobacteria[43,44], and for subsequent priming of adaptive immune effector cells[45]. Therefore, we investigated whether an increased rate of apoptosis in *Cd36^-/- ^*macrophages might explain the lower mycobacterial counts in these cells. By terminal deoxynucleotidyl transferase dUTP nick end labeling (TUNEL) assay, we found no differences in apoptosis between *Cd36^-/- ^*and *Cd36^+/+ ^*macrophages after 7 days of co-cultivation with *M. tb *or BCG (data not shown). Similarly, no difference was observed in caspase-3 activity between cultures of *Cd36^-/- ^*and *Cd36^+/+ ^*macrophages co-incubated with *M. tb *or BCG (data not shown). Furthermore, examination of spleen and liver tissue sections from *in vivo *infection experiments with BCG did not reveal any differences in the density of apoptotic cells (data not shown).

We also examined if there were differences in viability of infected *Cd36^-/- ^*macrophages compared to *Cd36^+/+^*, which could account for the observed lower mycobacterial counts in cultures of *Cd36^-/- ^*macrophages. Viability of macrophages, as determined by quantitative MTS assay, was equivalent over 7 days after infection with *M. tb *between *Cd36^-/- ^*and *Cd36^+/+ ^*macrophages (*p *= 0.578; data not shown).

### Production of reactive nitrogen and reactive oxygen intermediates is similar in *Cd36^+/+ ^*and *Cd36^-/- ^*macrophages

**Figure 6:**
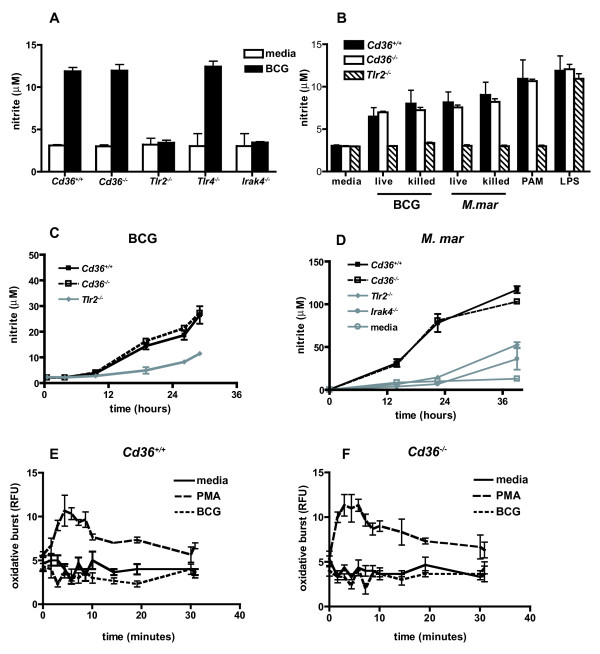
**Production of reactive nitrogen and reactive oxygen intermediates is similar in *Cd36^+/+ ^*and *Cd36^-/- ^*macrophages**. **A**. Nitric oxide production was measured using the Griess reaction in culture supernatant. In response to stimulation with BCG, nitric oxide production is TLR2 (*p < 0.0001) and IRAK-4 (*p < 0.0001) dependent, but independent of TLR4 (*p *= 0.16) and CD36 (*p *= 0.83). **B**. Similar nitric oxide production by *Cd36^+/+ ^*and *Cd36^-/- ^*macrophages in response to live and heat-killed BCG and *M. marinum*, as well as specific toll-like receptor ligands (Pam_3_CSK_4 _(PAM), a TLR2 ligand, and lipopolysaccharide (LPS); *p *> 0.05 for all comparisons *Cd36^+/+ ^vs Cd36^-/-^*). **C **and **D**. Similar kinetics of nitric oxide production over 36 hours in response to both BCG (**C**) and *M. marinum *(**D**). **E **and **F**. Similar oxidative burst in response to phorbol myristate acetate (PMA), and no significant response to BCG above baseline among *Cd36^+/+ ^*and *Cd36^-/- ^*macrophages.

The production of reactive nitrogen intermediates (RNI) and reactive oxygen intermediates (ROI) are effective intracellular killing mechanisms against microbial pathogens including mycobacteria[46,47]. We examined whether differences in nitric oxide production and/or oxidative burst in response to infection with mycobacteria might account for the restriction of mycobacterial growth in *Cd36^-/- ^*macrophages. Nitrite concentration was similar in the supernatant of interferon-γ (IFN-γ) activated *Cd36^+/+ ^*and *Cd36^-/- ^*macrophages stimulated with BCG for 24 hours (Figure 6A). Consistent with previous reports[48-50], nitric oxide production in response to mycobacterial challenge was toll-like receptor 2 (TLR2)- and interleukin-1 receptor-associated kinase 4 (IRAK-4)-dependent, but TLR4-independent in our experimental system (Figure 6A). Nitric oxide production in response to live and heat-killed BCG and *M. marinum*, as well as TLR2 and TLR4 specific ligands, was similar in *Cd36^+/+ ^*and *Cd36^-/- ^*(but not *Tlr2^-/-^*) macrophages (Figure 6B) and followed similar kinetics in both cell types (Figure 6C and 6D). Oxidative burst of *Cd36^+/+ ^*and *Cd36^-/- ^*macrophages, as determined by luminol-enhanced chemiluminescence assay, was similar in response to phorbol myristate acetate, and neither cell type produced significant reactive oxygen species above baseline in response to stimulation with BCG (Figure 6E and 6F), as previously described[51,52].

### Differences in mycobacterial susceptibility are not explained by polymorphisms of *Nramp1*

Because polymorphisms in the *Nramp1 *gene are known to affect host susceptibility to mycobacterial infection[53], we analyzed genomic DNA from *Cd36^+/+ ^*and *Cd36^-/- ^*mice for polymorphisms at this genetic locus. Comparing to C57BL/6 (BCG-sensitive) and 129/Sv (BCG-resistant) controls, both *Cd36^+/+ ^*and *Cd36^-/- ^*mice were found to carry characteristic C57BL/6 alleles (data not shown). Therefore, differences in mycobacterial susceptibility between the *Cd36^+/+ ^*and *Cd36^-/- ^*mice do not appear to be attributable to variation in the *Nramp1 *gene.

### Levels of TNF correlate with bacillary burden

**Figure 7:**
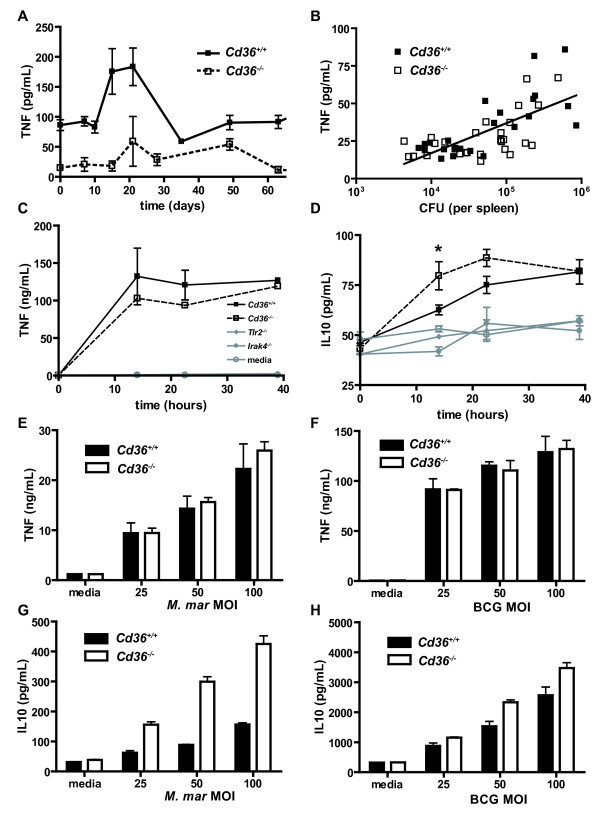
**Alterations in TNF and IL-10 production do not explain relative resistance of *Cd36^-/- ^*mice to mycobacterial infection**. **A**. Serum levels of TNF in *Cd36^+/+ ^*and *Cd36^-/- ^*mice rose after intraperitoneal infection (day 0) with *M. bovis *BCG, reaching a maximum after 14-21 days, then decreased. This trend paralleled the mycobacterial counts in organs of infected mice. TNF levels were higher overall (*p *< 0.001) in *Cd36^+/+ ^*mice (solid line) compared to *Cd36^-/- ^*mice (dashed line). Results are displayed as mean ± SEM, with each point representing 4 replicate mice. Data from one representative experiment of two are shown. **B**. Significant correlation (ρ = 0.596, *p *< 0.001) between splenic mycobacterial counts and serum level of TNF. **C**. Time course of TNF production by *Cd36^+/+ ^*and *Cd36^-/- ^*thioglycolate-elicited peritoneal macrophages co-incubated with *M. marinum in vitro *shows no difference between genotypes (*p *= 0.33). In contrast, TNF production by *Tlr2^-/- ^*(*p *< 0.0001) and *Irak4^-/- ^*(*p *< 0.0001) macrophages is markedly deficient. **D**. *Cd36^-/- ^*macrophages are not deficient in IL-10 production, and produce higher levels of IL-10 at some time points (* *p *= 0.040) following infection with *M. marinum*. **E **and **F**. TNF production in response to *M. marinum *(**E**) and BCG (**F**) is similar (*p *= 0.54 for *M. marinum*, *p *= 0.96 for BCG) between *Cd36^+/+ ^*and *Cd36^-/- ^*macrophages over a range of multiplicities of infection. **G **and **H**. IL-10 production in response to *M. marinum *(**G**) and BCG (**H**) was dose-dependent (*p *< 0.0001 for both) and was higher in *Cd36^-/- ^*macrophages (*p *< 0.0001 for *M. marinum*, *p *= 0.0003 for BCG). Cytokine levels were assayed in supernatant of macrophages in 96-well plates (200,000 adherent cells and 250 μl media per well) after 24 hours incubation. These findings were confirmed using bone-marrow derived macrophages (data not shown).

T_H_1 cytokine responses are critical for granuloma formation and control of mycobacterial infection[2], with TNF playing a central role[41]. We examined levels of TNF, IFN-γ, IL-10, IL-6, IL-12p70 and MCP-1 in the sera of mice infected with BCG to determine whether differential cytokine responses could explain the reduced BCG loads in *Cd36^-/- ^*mice. TNF concentrations rose to a maximum 2-3 weeks after infection and subsequently decreased (Figure 7A), in parallel with BCG counts in the spleen and liver. *Cd36^-/- ^*mice had lower TNF levels overall (*p *< 0.001). TNF levels were positively correlated with the BCG counts in the spleen (ρ = 0.596, p < 0.001, Figure 7B). Although it might be predicted that higher levels of T_H_1 cytokines would be associated with improved control of mycobacterial infection, *Cd36^-/- ^*mice exhibited lower levels of TNF, which in turn correlated with lower mycobacterial loads in infected organs. Rather than determining the course of infection, TNF levels appear to reflect the burden of infected macrophages in our mouse model.

In support of this explanation, we observed similar levels of TNF in the supernatant of *Cd36^+/+ ^*and *Cd36^-/- ^*thioglycolate-elicited peritoneal macrophages infected with mycobacteria *in vitro *(Figure 7C), which increased in a dose-dependent manner with increasing multiplicity of infection with both *M. marinum *(Figure 7E) and BCG (Figure 7F). These findings were replicated with bone-marrow derived murine macrophages (data not shown). In contrast, TNF production was reduced in *Tlr2^-/- ^*and *Irak4^-/- ^*macrophages compared to their wild type counterparts (Figure 7C).

### Altered IL-10 production does not account for enhanced antimycobacterial defenses in *Cd36^-/- ^*mice and macrophages

IL-10 is an immunomodulatory cytokine, known to inhibit macrophage antimycobacterial activity *in vitro*[54] and *in vivo*[55], as exemplified by the enhanced clearance of mycobacteria in IL-10 deficient mice[56-58]. Mycobacteria and their products, including the glycolipid AraLAM, induce IL-10 production by macrophages[59]. IL-10 production by macrophages in response to apoptotic cells is mediated by CD36 to a large extent[29,60]. Therefore, we hypothesized that the enhanced antimycobacterial activity of *Cd36^-/- ^*macrophages may be related to reduced production of IL-10. However, we observed significantly higher levels of IL-10 by *Cd36^-/- ^*macrophages over a time course of infection (Figure 7D) and over a range of multiplicities of infection with both *M. marinum *(Figure 7G) and BCG (Figure 7H). This finding was replicated using both thioglycolate-elicited peritoneal macrophages (Figure 7D, G and 7H) and bone-marrow derived macrophages (data not shown). In contrast, IL-10 production by *Tlr2^-/- ^*and *Irak4^-/- ^*macrophages was significantly reduced (Figure 7D). Furthermore, no difference in IL-10 levels was detected in the sera of *Cd36^-/- ^*mice relative to wild type controls over the course of experimental BCG infection (data not shown). Therefore, improved mycobacterial defenses in *Cd36^-/- ^*mice do not appear to be attributable to reduced production of IL-10 by macrophages.

Additionally, there were no significant differences observed in serum IFN-γ, IL-6, IL-10, IL-12p70 and MCP-1 levels in *Cd36^-/- ^vs Cd36^+/+ ^*mice up to 63 days after infection (data not shown).

### Reduced recovery of viable mycobacteria from *Cd36^-/- ^*macrophages using an *in vitro *model of cellular events involved in granuloma formation

**Figure 8:**
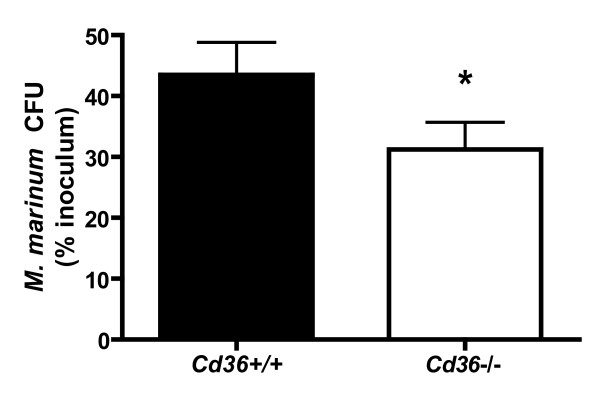
**Recovery of viable intracellular mycobacteria is reduced in *Cd36^-/- ^*macrophages in an *in vitro *model recapitulating cellular events in early granuloma formation**. Primary "prey" macrophages were incubated with *M. marinum *and washed to remove extracellular bacteria. Apoptosis was induced by overnight serum starvation, and was evidenced by increased caspase-3/7 activity (data not shown). Control macrophages (no apoptosis) were incubated in media containing 10% fetal bovine serum. Apoptotic primary prey macrophages containing *M. marinum *were gently scraped and co-incubated with uninfected secondary "predator" macrophages to allow phagocytosis of primary prey macrophages. After washing to remove extracellular apoptotic bodies and bacteria, cells were scraped and plated on mycobacteria culture media for CFU counts. The recovery of viable *M. marinum *was reduced in systems using *Cd36^-/- ^*(white bar) relative to *Cd36^+/+ ^*(black bar) macrophages (*p = 0.0026), suggesting a defect in the uptake of mycobacteria-laden apoptotic macrophages.

Based on studies using quantitative intravital imaging in transparent zebrafish infected with *M. marinum*, macrophage turnover within the granuloma contributes to early mycobacterial growth and dissemination[4]. Arriving macrophages efficiently find and phagocytose infected macrophages undergoing apoptosis, leading to expansion of infected macrophages and bacterial numbers[4]. Given the role of CD36 in the uptake of apoptotic cells by macrophages[29], this observation may account for the decreased mycobacteria loads observed in *Cd36^-/-^*mice. We therefore designed an *in vitro *system to recapitulate the cellular events of *M. marinum *internalization, apoptosis of infected macrophages, and phagocytosis by secondary uninfected macrophages. Peritoneal macrophages from *Cd36^+/+ ^*or *Cd36^-/- ^*mice were plated and infected with *M. marinum *(MOI = 10:1). Apoptosis of the infected macrophages was induced by serum starvation, as demonstrated by elevated caspase-3/7 activity[39] (data not shown). Then, the infected apoptotic "prey" macrophages were gently scraped and co-incubated with healthy uninfected "predator" macrophages. After incubation at 37°C for 3 hr to allow internalization of prey macrophages, cells were carefully washed to remove extracellular apoptotic bodies and bacteria. Cells were lysed and plated on mycobacterial culture media for CFU counts. The fraction of viable mycobacteria recovered was reduced in *Cd36^-/- ^*compared to *Cd36^+/+ ^*predator macrophages (*p *= 0.0026; Figure 8). These observations are consistent with the known role of CD36 as a receptor for apoptotic cell uptake on phagocytic cells[29], together with their bacterial contents. Collectively, these results suggest that CD36 participates in granuloma turnover, and contributes to the expansion of the intracellular mycobacterial pool.

## Discussion

Host genetic factors play a major role in influencing the severity and the ultimate outcome of tuberculosis. In this study, we have identified CD36 as a potentially important determinant of host susceptibility to mycobacterial infection. We demonstrated that CD36 deficiency confers relative resistance to mycobacterial infection. This conclusion was supported by both *in vivo *experimental model infections and *in vitro *cell culture infection approaches.

We used a well-established murine model of disseminated mycobacterial infection and demonstrated that disruption of the *Cd36 *gene confers an altered susceptibility phenotype. In this experimental murine model, systemic BCG infection is ultimately controlled with the induction of antigen-specific immunity, similar to human tuberculosis[17,40,48]. In our studies, *M. bovis *BCG counts in the spleens and livers of infected mice rose to a maximum after 2 weeks, and subsequently declined to nearly undetectable levels with the induction of adaptive immunity (Figure 1). Splenomegaly and granulomatous infiltrates in the spleen and liver have been previously described in murine *M. bovis *BCG infection[19], as observed in our experiments (Figure 2). We used three species of mycobacteria (*M. tb*, *M. marinum *and BCG) for *in vitro *experiments in order to model distinct aspects of tuberculosis pathogenesis and demonstrate the generalizability of these findings. In addition to sharing many known virulence factors with *M. tb*, *M. marinum *showed growth restriction similar to M. tb within *Cd36^-/- ^*macrophages (Figure 3), and was therefore used in subsequent assays as a model organism. *M. marinum *offers several technical advantages including a rapid growth rate and minimal biohazard risk. Where feasible, we replicated our findings with BCG to demonstrate generalizability across different mycobacterial species. However, because BCG forms large aggregates when cultured *in vitro*[61], it was not suitable for all experiments, particularly imaging studies which required suspensions of single organisms.

Disruption of the *Cd36 *gene affected the early and peak mycobacterial burdens, but did not appear to impact the ultimate clearance of the organism (Figure 1), consistent with the role of CD36 as a receptor predominantly functioning in innate immunity[62]. Likewise, a recent study demonstrated a limited role for CD36 in controlling the outcome of *M. tuberculosis *pulmonary infection, with no survival difference and a modest effect on lung mycobacterial loads and granulomas only observed at early time points following intranasal challenge[63]. Further evidence of intact adaptive immune mechanisms includes the universal survival of *Cd36^-/- ^*mice, and the microscopic structure of granulomas, which were morphologically normal albeit reduced in number, commensurate with the reduced mycobacterial loads observed in these mice (Figure 2). As a macrophage cell surface receptor involved in the uptake of apoptotic cells[29], CD36 may participate in the cellular dynamics within the granuloma. Recent landmark studies have shown that mycobacteria promote and exploit granuloma formation for the establishment of infection[4]. After internalization of mycobacteria, macrophages undergo apoptosis, and are phagocytosed by newly recruited macrophages followed by egress of these cells to seed new granulomas[4]. We modeled this process *in vitro *and found reduced recovery of viable intracellular *M. marinum *from *Cd36^-/- ^*macrophages exposed to apoptotic macrophages containing mycobacteria (Figure 8). These data link our *in vivo *findings with recent insights into tuberculosis pathogenesis, suggesting that CD36 may play a role in the cellular events co-opted by mycobacteria during the establishment and dissemination of infection.

Consistent with results of *in vivo *studies, cell culture infection experiments demonstrated that CD36 deficiency limits intracellular replication of mycobacteria in macrophages (Figure 3). The mechanism underlying the restricted replication of mycobacteria in *Cd36^-/- ^*mice or macrophages is presently unclear; nevertheless, we have excluded differences in early mycobacterial uptake, macrophage cell viability or apoptosis, production of reactive nitrogen and oxygen species, *Nramp1 *gene, and selected cytokine responses among possible explanations. Our finding that CD36 was not required for mycobacterial internalization was similar to one recent report involving BCG uptake by murine peritoneal macrophages[63], but contrasts with an earlier study that implicated the *Drosophila *homologue of CD36 in *M. fortuitum *uptake[35]. Of note, however, transfection of murine *Cd36 *into HEK293 cells did not enhance uptake of *M. fortuitum *in the latter study[35], consistent with our and others' findings using murine macrophages[63]. Another possibility is that inherent differences in the properties of the mycobacterial species used in the earlier and the current studies may account for this discrepancy.

The restricted growth of mycobacteria within *Cd36^-/- ^*macrophages might be explained by impairment of mycobacterial immune evasion strategies that take advantage of CD36. Detailed structural studies of the mycobacterial cell wall lipomannans (LMs) have demonstrated that diacylated LMs inhibit LPS-induced inflammation by murine macrophages [64]. Intriguingly, CD36 is a sensor of diacylglycerides[31] from a broad range of pathogens[30,33,65] and may be the host receptor through which diacylated LMs suppress macrophage function. Alternatively, this observation may be explained by participation of CD36 in Toll-like receptor signaling. CD36 is known to associate with the TLR2/6 heterodimer on the cell surface[30,31], and may participate in TLR2-dependent immunosuppressive signaling pathways in the context of mycobacterial infection[66-69]. In our experiments, nitric oxide, TNF and IL-10 production in response to mycobacteria were TLR2 dependent; however, CD36 did not appear to participate in these processes. Nonetheless, a role for CD36 in other TLR2 mediated mycobacterial evasion mechanisms cannot be excluded.

Alterations in cytokine profile did not appear to explain CD36-mediated differences in mycobacterial control *in vivo *and *in vitro*. Reduced TNF in the sera of infected *Cd36^-/- ^*mice around the peak of infection appears to reflect the reduced mycobacterial stimulus rather than acting as a mediator of antimycobacterial defenses. *In vitro*, TNF production in response to live BCG and *M. marinum *was no different between *Cd36^-/- ^*and *Cd36^+/+ ^*macrophages, consistent with one previous report[63]. Other T_H_1 cytokines, IFN-γ and IL-12, were not significantly different in BCG-infected *Cd36^-/- ^*and *Cd36^+/+ ^*mice. Likewise, anti-inflammatory IL-10 levels were not significantly different *in vivo*, and were increased *in vitro *in *Cd36^-/- ^*murine macrophages pre-stimulated with IFN-γ. We speculate that the latter finding may suggest a role for CD36 in the previously described IFN-γ inhibition of TLR2-induced IL-10 expression in the context of mycobacterial infection[70]. However, elevated levels of IL-10 do not appear to explain the reduced mycobacterial counts in *Cd36^-/- ^*macrophages, given the known inhibitory role of IL-10 on mycobacterial control[55,58].

Our observations that deficiency of CD36 reduces the susceptibility of mice *in vivo *and of murine macrophages *in vitro *to mycobacterial infection are novel and somewhat unexpected, given the roles of CD36 in host defense against pathogens such as *S. aureus *and other bacteria[30,32], as well as *P. falciparum*[33]. Although the underlying molecular mechanisms remain unclear and require further investigation, our study nevertheless suggests a unique role of CD36 in host susceptibility to tuberculosis. Within this context, it is interesting to note that a *CD36^-/- ^*genotype occurs with relatively high frequency in African, Japanese and other Asian populations[71,72], although the evolutionary advantage of this putative balanced polymorphism is unknown. First described in patients refractory to platelet transfusion[73], this genotype has subsequently been associated with susceptibility to a variety of metabolic diseases[74]. Recent population and family-based studies have not associated *CD36 *gene polymorphisms with severe malaria phenotypes and it has been suggested that CD36 deficiency alleles may be maintained in human populations through selection pressure via a prevalent infection other than malaria[75,76]. If our observations are subsequently confirmed in human infection, it is conceivable that *M. tb*, being highly prevalent and virulent, could in part account for the persistence of CD36 deficiency in populations from tuberculosis-endemic regions.

## Conclusions

In summary, our findings indicate a novel role for CD36 in host response to mycobacterial infection and suggest that future population-based studies to examine the relationship between CD36 deficiency and susceptibility to tuberculosis would be of interest.

## Competing interests

The authors declare that they have no competing interests.

## Authors' contributions

MH designed, executed, and analysed *in vivo *and *in vitro *experiments and wrote the manuscript. XL executed *in vivo *and *in vitro *experiments. MC planned early *in vitro *experiments. AD performed cytokine assays for *in vivo *experiments. CF performed and interpreted flow cytometry experiments. GMO analyzed genomic DNA for the *Nramp1 *gene. WCL participated in the design of the study and helped to draft the manuscript. JL conceived of the study, participated in its design and helped to draft the manuscript. KCK conceived of the study, participated in its design and helped to draft the manuscript. All authors have read and approved the final manuscript.

## Pre-publication history

The pre-publication history for this paper can be accessed here:

http://www.biomedcentral.com/1471-2334/10/299/prepub
